# A Case of Pneumonia Produced by Asphyxia

**Published:** 1843-06

**Authors:** G. W. Bayless

**Affiliations:** Demonstrator of Anatomy in the Louisville Medical Institute


Art. IV.—*A Case of Pneumonia produced by Asphyxia.* By G. W. Bayless, M. D., Demonstrator of Anatomy in the Louisville Medical Institute.

On the 17th of October last, I was notified to attend an examination of the body of Brayer, at the Louisville Marine Hospital. He was thirty-one years of age, had been laborer and boatman by occupation, and had generally enjoyed good health. It was said that both his father and mother had died of phthisis, but that no symptom of the disease had ever manifested itself in him.

According to the account given by those who took him to the Hospital, he fell overboard from a steamboat at the Vicksburg landing, about the 26th of September. After remaining some minutes under water, he was taken out, to all appearances lifeless; he lay totally insensible for several minutes, but, by some means or other, was finally resuscitated. He remained on the boat, which soon left for Louisville; and as he said himself, he was taken sick immediately. On the way, he had a dull pain in the left side, accompanied with cough and expectoration of some kind. He took no medicine on the way; the trip occupying about ten days. At the time that he entered the Hospital he was still laboring under the symptoms above enumerated, the expectoration being an opaque yellow mucus, sometimes presenting a rusty appearance. The ordinary physical signs of pneumonia, added to these functional ones, served to render the diagnosis of the case very plain. The treatment to which he was subjected in the Hospital, consisted of general and local bloodletting, blistering, the antimonial, and finally, I believe, the mercurial treatment. The above concise history of the case, is substantially that given by the attending physician, Professor Caldwell, just prior to the examination.

AUTOPTICAL APPEARANCES SIX HOURS AFTER DEATH.

*Thorax.*—Deposition of recent and imperfectly organized coagulable lymph over a great portion of the anterior part of the left lung, producing slight adhesion. On the posterior part of the same lung there was also copious deposition of lymph, likewise recent, but more perfectly organized, and producing a stronger adhesion. Very near the apex of this lung, on its anterior part, was a flattened and somewhat circular cavity, about an inch and a half in diameter, which contained about a teaspoonful of pus. It was essentially a pleuritic abscess, and its walls consisted of pretty firm false membrane. On the posterior part of the right lung there was likewise a deposition of recent lymph, but not so copious as on the left side, and the adhesion was not so strong. The right lung was greatly engorged with blood throughout, and there was a circumscribed portion about the size of the fist, near the middle of its back part, in a state of hepatization. Some small black spots, of an apoplectic appearance, were also seen in the same region. The left lung presented, in its various parts, all of the three stages of pneumonia. Its anterior and inferior portion presented the deep engorgment with blood flowing freely upon incision, and the sligh increase of solidity, characteristic of the first stage. Another part was in a state of hepatization; and the remainder presented the softening and purulent infiltration of the tissue of the lung, which characterizes the third stage.* At the upper and back part of this lung, about four inches from the top, was a cavity about an inch in diameter, which was filled with dark venous and fluid blood. On three sides it was surrounded by a dark red, semi-solid substance, and on the other, by the pleura and false membrane only It was a well marked instance of interstitial apoplexy. Several small spots of the same kind were also seen in the same region. The heart was in a healthy condition.

*Abdomen.*—No disease of any of its viscera.

*Head.*—There having been no indications of disease in this cavity it was not examined.

---

* This is according to Laennec and others. Stokes divides the disease into five stages.

In presenting this case, it is by no means my purpose merely to show the ravages produced by inflammation on the surface and in the substance of the lungs; but to exhibit a somewhat rare instance of inflammation following asphyxia. I suppose that in this case, as is common in asphyxia, an accumulation of blood took place in the whole extent of the circulatory apparatus for venous blood; that from suspended respiratory movements, or from the failure of the unchanged venous blood (the air being excluded) to afford the proper stimulus to the radicles of the pulmonary veins, or from both these causes together, the accumulation commenced in these veins, that it then took place successively in the branches of the pulmonary artery, in the artery itself, the right ventricle, the right auricle, venæ cavæ, &c. Under this state of things, the right ventricle would necessarily (the exclusion of air continuing, and death not yet having taken place in the brain from its want of supply of arterial blood) force on its contents into the already distended pulmonary arteries, and the radicles of the pulmonary veins, until there should be produced a very great engorgement, if not absolute rupture, of them. The individual being then removed from the circumstances which brought on this condition of things, and respiration re-established, this engorgement of blood in the lungs would be dissipated slowly, and prove a source of irritation that would cause the development of all the inflammation and its consequences that we have seen. The congestion in this case, as the immediate consequence of asphyxia, was altogether different from that in ordinary inflammation in other parts of the body; and the inflammation was effected in a different manner. When inflammation is setting up in other organs, or in the lungs from other causes, the congestion is in the nutritious vessels of the part; but in this case there was a deep engorgement of the pulmonary arteries and veins, as the immediate consequence of asphyxia, and this, by mechanical distention and consequent irritation, invited a secondary congestion in the ramifications of the bronchial arteries, which last led on to the inflammation and its consequences. As to the mode in which the various consequences of inflammation, which we found, resulted in this particular case, it would of course be superfluous to speak. They all took place in accordance with the well known laws of that morbid action.

It may be asked, did not the individual take cold, and was not the pneumonia produced, as ordinarily, by the impress of that agent? I reply, that two circumstances in the history of the case induce me to think not. First, that the accident occurred at a season of the year when pneumonia is not readily produced in a stout man by such an exposure to cold. Secondly, that he was taken sick *immediately*, which would not have been the case if cold had been the agent in the production of the disease; whereas the congestion of the pulmonary vessels is quite sufficient to account for his immediate indisposition.

May, 1843.

